# Author Correction: Wogonin reversed resistant human myelogenous leukemia cells via inhibiting Nrf2 signaling by Stat3/ NF-κB inactivation

**DOI:** 10.1038/s41598-021-91964-z

**Published:** 2021-06-11

**Authors:** Xuefen Xu, Xiaobo Zhang, Yi Zhang, Lin Yang, Yicheng Liu, Shaoliang Huang, Lu Lu, Lingyi Kong, Zhiyu Li, Qinglong Guo, Li Zhao

**Affiliations:** 1 State Key Laboratory of Natural Medicines, Jiangsu Key Laboratory of Carcinogenesis and Intervention, China Pharmaceutical University, 24 Tongjiaxiang, Nanjing, 210009 People’s Republic of China; 2 State Key Laboratory of Natural Medicines, Department of Natural Medicinal Chemistry, China Pharmaceutical University, 24 Tongjiaxiang, Nanjing, 210009 People’s Republic of China

Correction to: *Scientific Reports* 10.1038/srep39950, published online 02 February 2017

This Article contains errors, which the authors’ institution confirmed arose through mistakes in figure assembly.

In Figure 2E, the images for the untreated group were inadvertently used for the LPS/wogonin treatment. The correct Figure 2 appears below as Figure 1.

**Figure 1:**
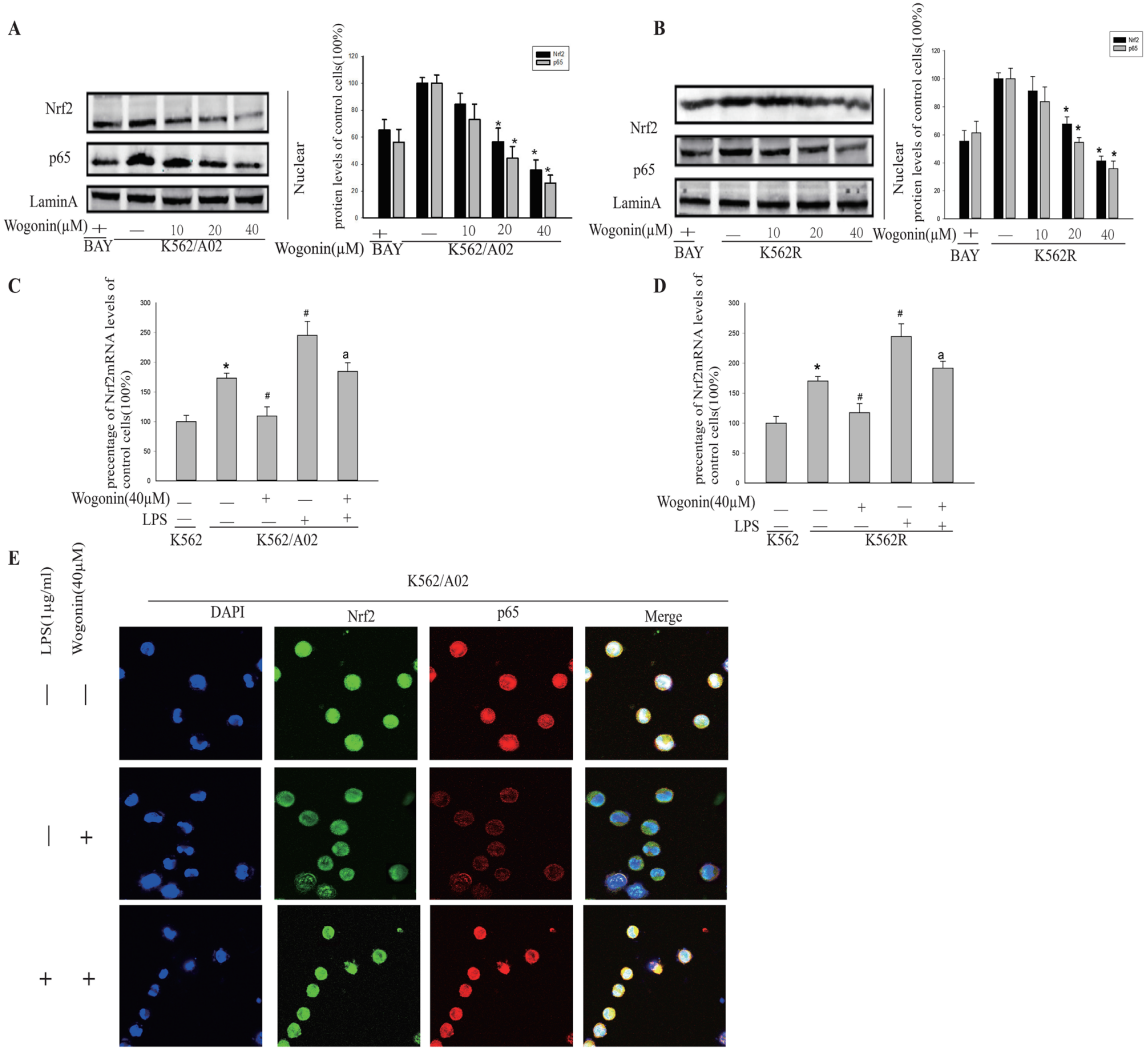
A correct version of original figure 2.

In Figure 6B, images were duplicated between the control group and Nrf2siRNA + ADR group, and between the Nrf2 SiRNA group and ADR (10 μM) group. This experiment has been repeated, and an updated Figure 6, with the new data appears below as Figure 2.

**Figure 2:**
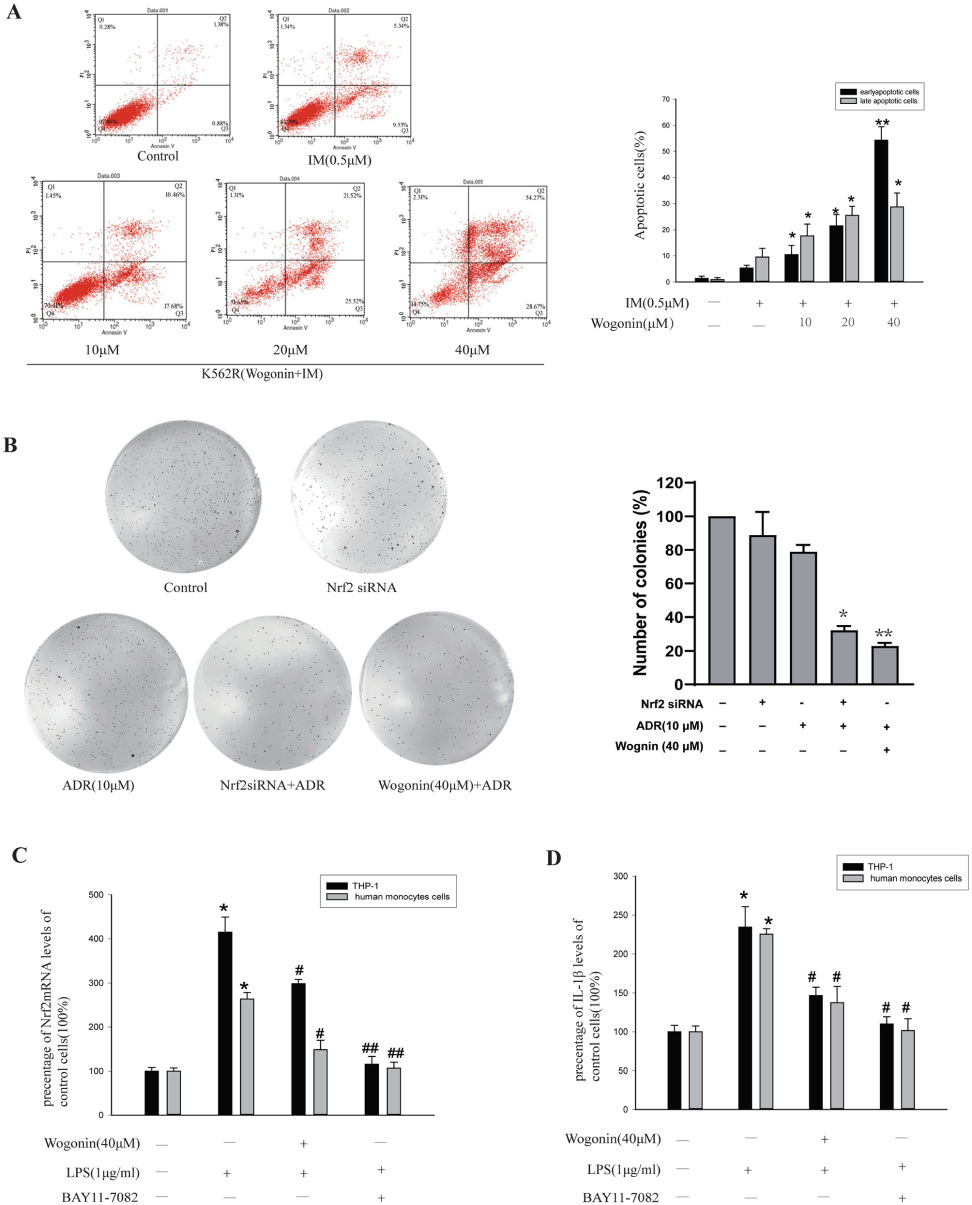
A correct version of original figure 6.

In Figure 8E, the incorrect image was used for the Nrf2/wogonin K562/A02 panel, p-Stat3/wogonin 562/A02, and for the p65/untreated K562/A02 panel. A revised version of Figure 8 appears below as Figure 3, with new data collected from the original samples.

**Figure 3:**
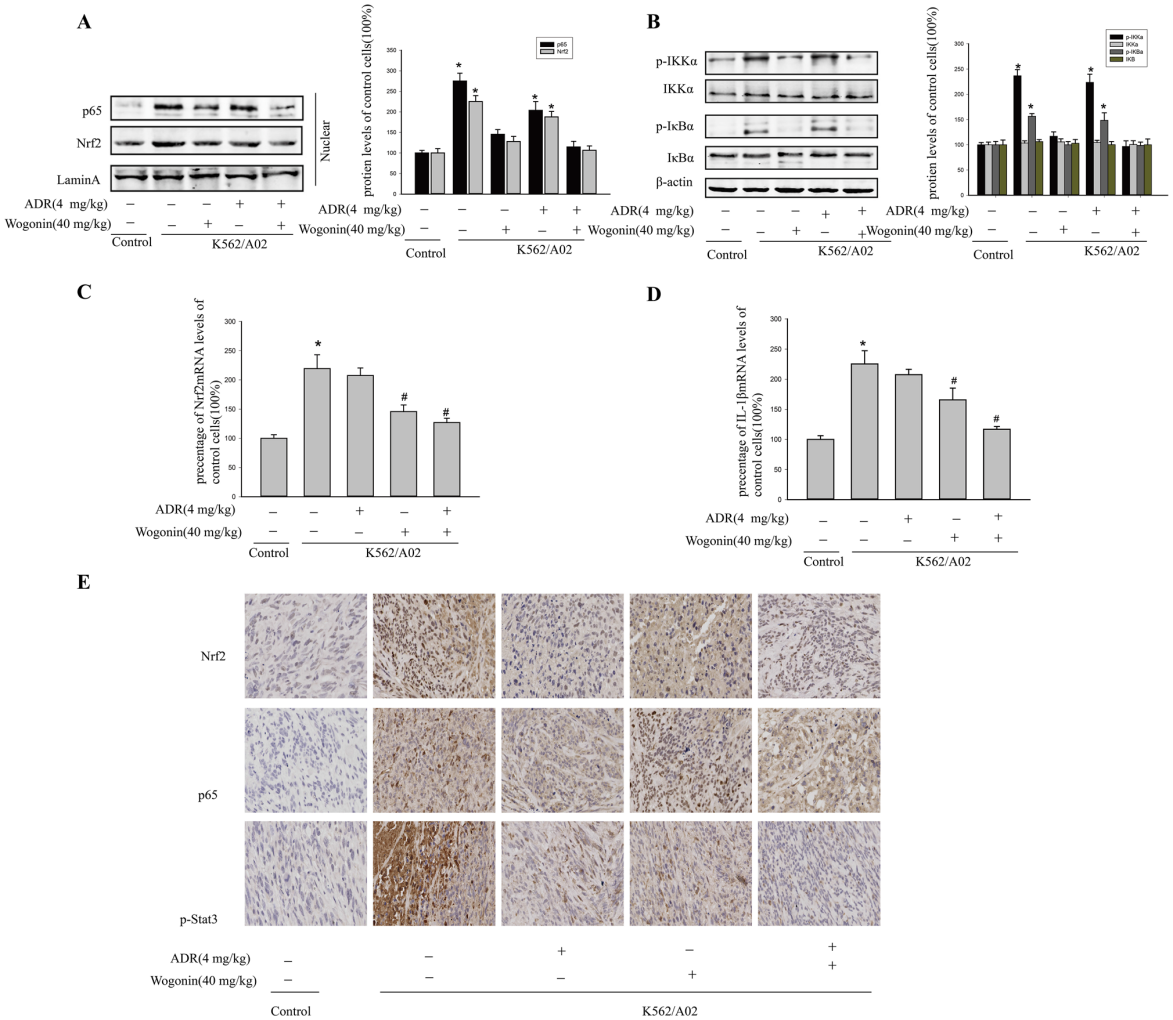
A correct version of original figure 8.

The full blots were not provided in the original article, and these have now been provided in the Supplementary Information file below.

These corrections do not change the conclusions of the paper.

## Supplementary Information

Full blots for the paper.

