# Supplementary material for: Author Correction: Wogonin reversed resistant human myelogenous leukemia cells via inhibiting Nrf2 signaling by Stat3/ NF-κB inactivation
Source: Sci Rep. 2021 Jun 11;11:12746. doi: 10.1038/s41598-021-91964-z (PMC8196212; doi:10.1038/s41598-021-91964-z)

Supplementary Information file containing full blots for the paper

Wogonin reversed resistant human myelogenous leukemia cells via inhibiting Nrf2 signaling by Stat3/ NF-κB inactivation.

XuefenXu[*], XiaoboZhang[*], Yi Zhang, LinYang, Yicheng Liu, Shaoliang Huang, Lu Lu, Lingyi Kong, Zhiyu Li, QinglongGuo & Li Zhao

Figure 1C-p-ERK

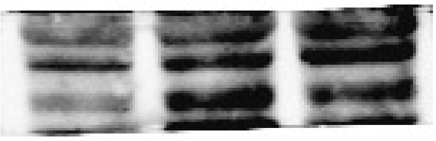

Figure 1C-β-actin

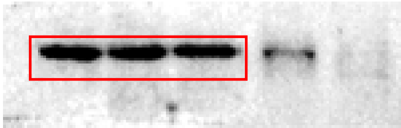

The red box means the raw blots of final result.

Figure 1D-p-ERK

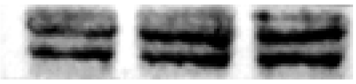

Figure 1D-β-actin

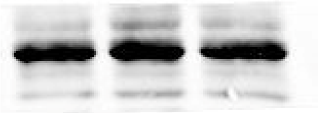

Figure 1F-p- IκBα

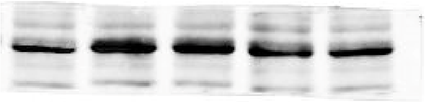

Figure 1F-IκBα

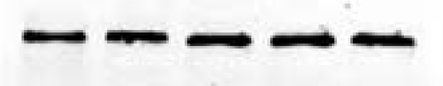

Figure 1F - p-IKKα

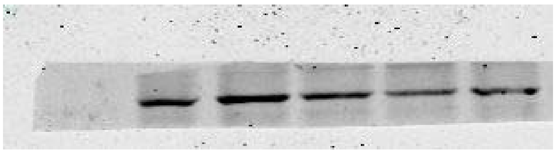

Figure 1F - IKKα

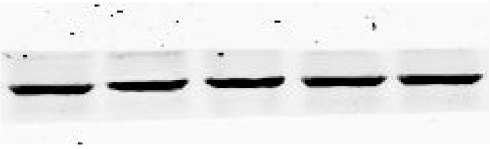

Figure 1F - β-actin

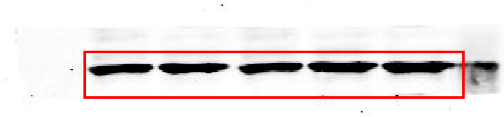

The red box means the raw blots of final result.

Figure 1G - p-IκBα

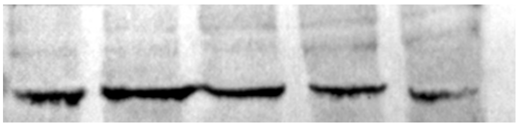

Figure 1G - IκBα

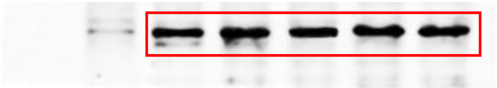

The red box means the raw blots of final result.

Figure 1G - p-IKKα

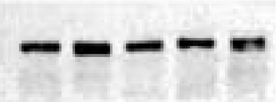

Figure 1G - IKKα

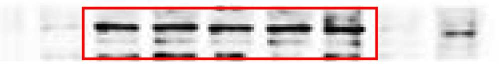

The red box means the raw blots of final result.

Figure 1G - β-actin

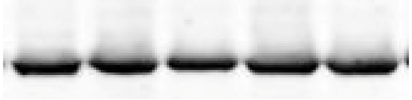

Figure 2A- Nrf2

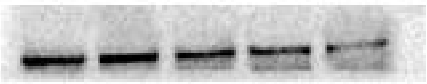

Figure 2A- p65

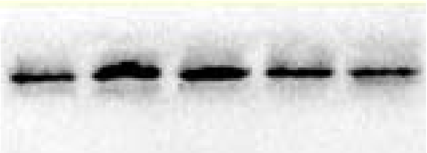

Figure 2A- Lamin A

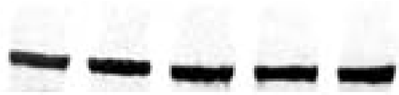

Figure 2B- Nrf2

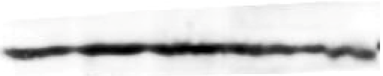

Figure 2B- p65

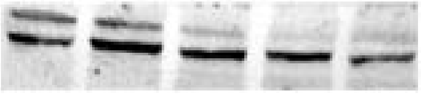

Figure 2B- LaminA

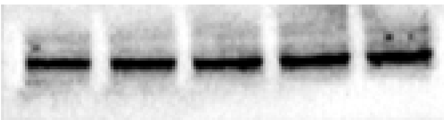

Figure 3A- EMSA

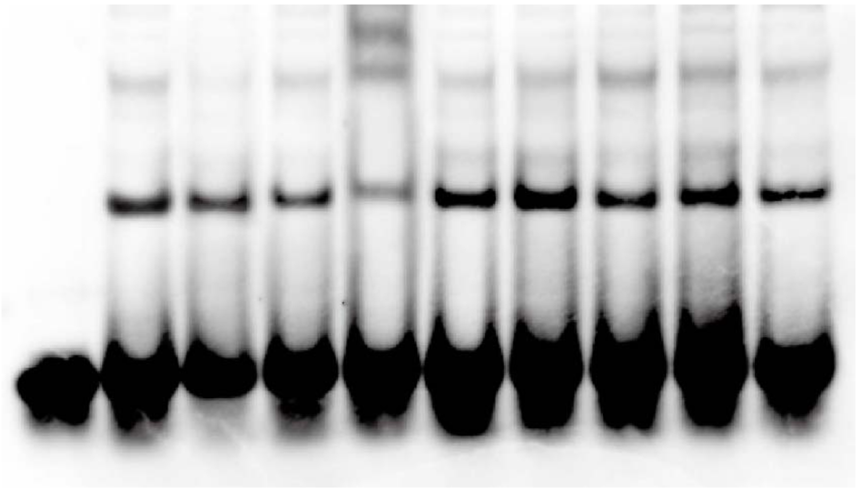

Figure 3B - EMSA

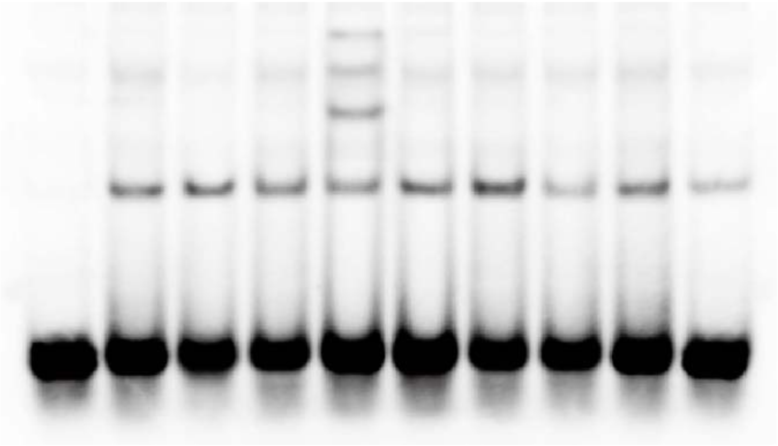

Figure 3C - p50

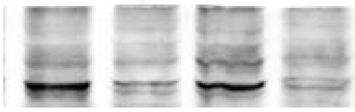

Figure 3C - p65

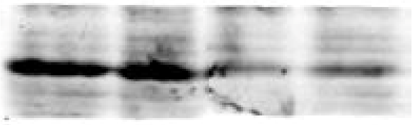

Figure 3C - β- actin

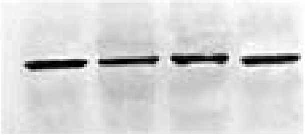

Figure 3C - EMSA

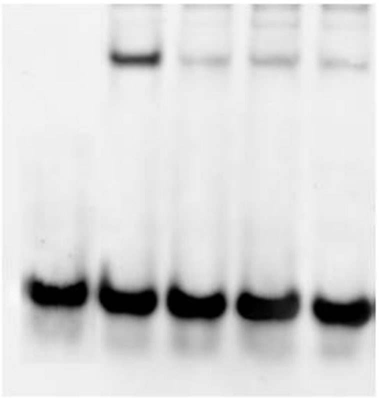

Figure 3D- p50

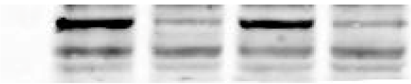

Figure 3D- p65

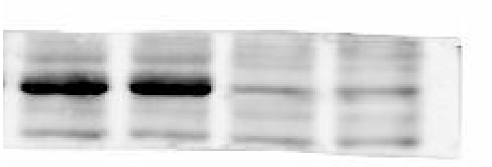

Figure 3D- β- actin

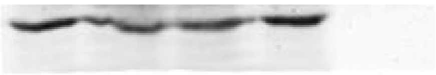

Figure 3D- EMSA

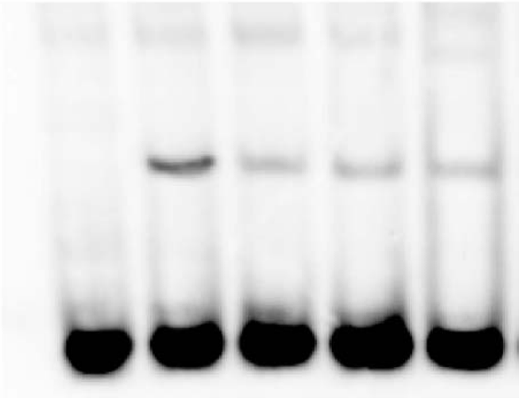

Figure 4F- EMS

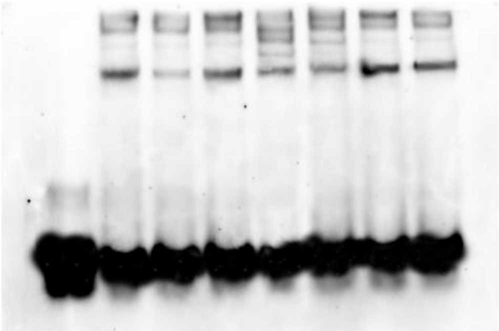

Figure 5A- p- Stat3

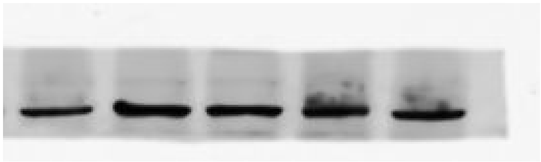

Figure 5A-Stat3

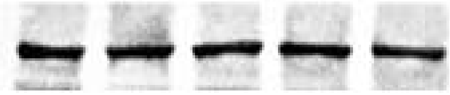

Figure 5A-p-BCR-ABL

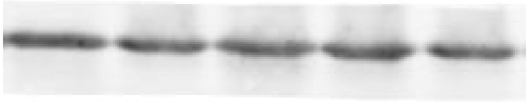

Figure 5A-β-actin

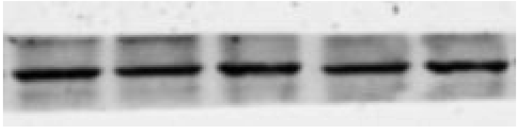

Figure 5B-p65

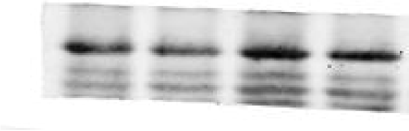

Figure 5B-p-Stat3

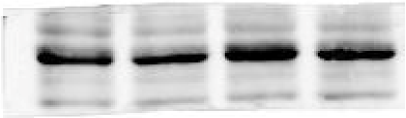

Figure 5B-LaminA

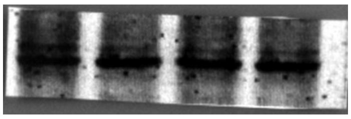

Figure 5C-EMSA

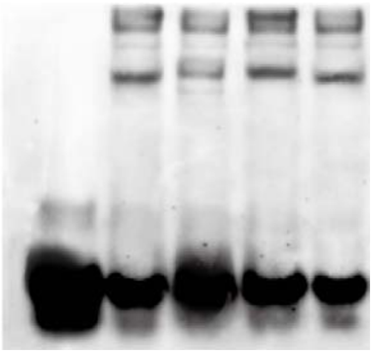

Figure 8A- p65

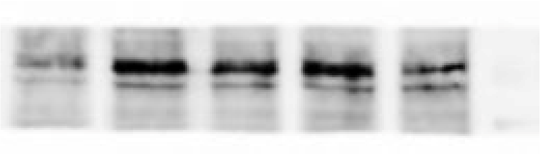

Figure 8A- Nrf2

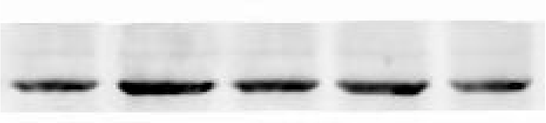

Figure 8A- LaminA

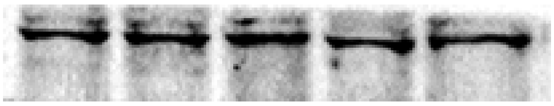

Figure 8B - p-IKKα

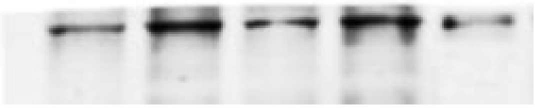

Figure 8B - IKKα

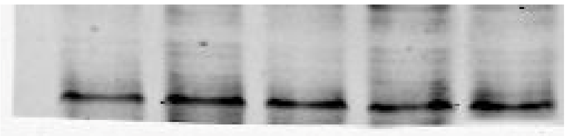

Figure 8B - p-IκBα

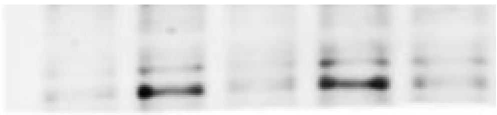

Figure 8B - IκBα

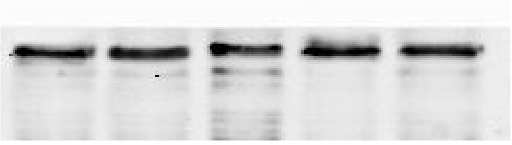

Figure 8B - β-actin

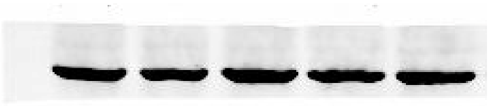

Supplement: Supplementary file 1 — Full blots for the paper. [file 41598_2021_91964_MOESM1_ESM.pdf]
